# Public perceptions about the invasive pampas grass, *Cortaderia selloana*: a case study of environmentally conscious citizens in Southern Europe

**DOI:** 10.1007/s10530-023-03025-3

**Published:** 2023-03-28

**Authors:** Mónica Roldão Almeida, Elizabete Marchante, Hélia Marchante

**Affiliations:** 1 Polytechnic Institute of Coimbra, Coimbra Agriculture School, Bencanta, 3045-601 Coimbra, Portugal; 2 Present Address: Centre for Functional Ecology, Department of Life Sciences, University of Coimbra, Calçada Martim de Freitas, 3000-456 Coimbra, Portugal

**Keywords:** Invasive species, Online survey, Questionnaire, Public education, Social media, Iberian Peninsula

## Abstract

*Cortaderia selloana* (pampas grass), native to South America, is a widespread invasive plant in several regions of the World, including the south of the Atlantic Arc (Europe), where it has been used as an ornamental species. Citizens may help to spread it, e.g., planting it in their gardens, but on the other hand, when they are aware of its invasiveness, can contribute to control it and prevent its spread. An online survey was performed to better understand the perception and knowledge of Portuguese and Spanish citizens, regarding pampas grass. The influence of education and occupation, along with age, gender and country of residence, on the knowledge and perceptions of respondents was analysed. The questionnaire was answered by 486 and 839 citizens in Portugal (PT) and Spain (ES), respectively. Most respondents were between 41 and 64 years old, mostly women in Portugal and equally women and men in Spain, with higher education and working mostly in the services sector. The majority of respondents in both countries recognized the plant, knew it is invasive and were able to name it, alerting to a possible bias of the target audience toward citizens already aware of the invasiveness of the pampas grass. Fewer respondents were aware of the legislation that limits its use, and most were unable to identify particular characteristics of the species. The results showed that respondents' occupation in PT and education in ES influenced their knowledge and perception about pampas grass. This study confirms that education and raising awareness regarding invasive species is of utmost importance, as respondents identified academic training and projects with a strong focus on public awareness as the main sources of knowledge regarding pampas grass. Better informed citizens can be part of the solution rather than part of the problem, especially regarding invasive species with such ornamental interest as pampas grass.

**Supplementary Information:**

The online version contains supplementary material available at 10.1007/s10530-023-03025-3.

## Introduction

Invasive alien species (IAS) pose a major threat to biodiversity worldwide, with huge impacts on both human health and the economy (Mazza et al. 2014; IPBES 2019; Diagne et al. 2021). The average annual costs of biological invasions (including both damage and management) can be over US$160 billion worldwide, but this figure is clearly underestimated, as the true costs of some of the world’s 100 worst invasive species remain unknown and the costs of invasive plants are frequently underdocumented (Cuthbert et al. 2021; Diagne et al. 2021; Novoa et al. 2021). Damage cost is an order of magnitude higher than management expenditures, stressing the need for management actions and international policy agreements to reduce the burden of invasive species (Diagne et al. 2021). Impacts on human health from invasive plants include direct exposure, pathogens, disease vectors, toxins, contamination of edible foodstuff, and allergens; additionally, these species may promote indirect implications, such as impacts on the environment and on ecosystem services, which in turn may affect human health (Kumar Rai and Singh 2020). Actions to prevent and mitigate the impacts of invasive species require the involvement and support of the whole society, but despite increasing investment in raising public awareness (Marchante and Marchante 2016; Verbrugge et al. 2021), many citizens, especially outside the scientific community, are still unaware of IAS consequences and do not always support their management (Novoa et al. 2017; Ricciardi and Ryan 2018; Potgieter et al. 2019; Cordeiro et al. 2020; Kowarik et al. 2021).

*Cortaderia selloana* (Schult. & Schult.f.) Asch. & Graebn., commonly known as pampas grass, is native to South America, namely southern Brazil, Uruguay and Argentina. This is a gynodioecious species, with hermaphrodite plants producing viable seeds in small amounts and being essentially pollen donors, implying that both hermaphrodite and female plants need to be relatively close so that widespread reproduction can occur (Connor 1973). Female specimens were initially introduced worldwide as ornamental plants. After the introduction of hermaphrodite plants, the species start spreading mostly in urban zones, disturbed areas, estuaries, dune systems and along roads, highways and rails; later it spread to new areas and nowadays it invades also natural habitats (Basnou 2009). The particular breeding system, and the fact that only female plants were initially propagated and commercialized (Robacker and Corley 1992; Grounds 1998), may have contributed to the apparent ignorance of many people about its invasiveness (author’s personal observation). In addition to planting pampas grass in their gardens, citizens can also be responsible for their unintentional dispersal whenever they collect inflorescences for interior decorations. Nowadays, pampas grass is a widespread invasive species in California (Lambrinos 2001), New Zealand (Than and Aliaga 2010), South Africa (Robinson 1984) and southern Europe (Basnou 2009) particularly in Portugal (Marchante et al. 2014) and Spain (Gobierno de Cantabria 2017). *Cortaderia selloana* can change significantly the natural and seminatural habitats (*e.g.,* by decreasing species richness, diversity and plant cover), threatening natural vegetation (Domènech et al. 2006; Gallastegui and Prieto 2010), achieving the highest overall impact score when compared with other alien grasses (Nkuna et al. 2018). Pampas grass can also trigger pollen allergies or respiratory distress in sensitive people (Fernández et al. 2017), extending the period of grass allergies in Northern Spain to about three months every year (Rodríguez et al. 2021). It can also cause skin cuts due to its sharp leaves (Dehnen-Schmutz 2015; González et al. 2020). In Portugal and Spain, the species is included in the national lists of invasive species since 2019 (Ministério do Ambiente 2019) and 2013 (MAGRAMA 2013), respectively. The species is under evaluation to be included in the list of invasive alien species of European Union concern (EU Regulation 1143/2014, The European Commission 2014), with *Cortaderia jubata*, a closely related species, already on that list.

Understanding public perception of invasive species is important for better planning their management (Shackleton et al. 2019b), not only because citizens may contribute to preventing their establishment and spread (by not using them), but also to support or even promote their control. Therefore, in the last decade, several studies have analysed public and stakeholders’ perceptions, engagement and knowledge about invasive species (Rai et al. 2012; Verbrugge et al. 2013; Shackleton et al. 2019a, b; Vaz et al. 2019; Cordeiro et al. 2020; Kowarik et al. 2021; Sosa et al. 2021). Often used as a decorative and ornamental species, people consider pampas grass to be beautiful while unintentionally disregarding its impacts as an invasive plant. In fact, activities that promote the species (*e.g.,* https://pt.esdemgarden.com/grass-of-pampas-cortaderia-selloana-9405) and observations during public awareness activities point out that part of the Iberian population is still unaware of its invasiveness. However, the perception of citizens was not properly explored regarding this species, although it is particularly relevant when it comes to a species frequently used by citizens and that is so easily wind-dispersed through carefree use. The LIFE project “Stop *Cortaderia*—Urgent measures for controlling the spread of pampas grass (*Cortaderia selloana*) in the Atlantic area” (2018–2022), set as one of its main goals to raise public awareness about pampas grass in Portugal, Spain and France, among other tasks. In Portugal, the citizen-science platform INVASORAS.PT (Marchante et al. 2017) has also been working since 2013 to raise citizens' awareness regarding invasive plants, including pampas grass. In this context, after developing numerous activities to raise public awareness within these two projects, *e.g.,* public talks, training sessions and short courses, exhibitions, technical seminars, and dissemination on social media, a survey was performed to analyse the perceptions and knowledge of pampas grass in Portugal and Spain.

## Methods

###  Target public

Our target audience was a subgroup of the Portuguese and Spanish population with access to the internet. Portugal and Spain were selected since these are the main territories included in the abovementioned projects. Although internet-based surveys may limit the outreach as the internet is not available to the entire population, in 2021 ca. 78 and 93% of the Portuguese and Spanish populations, respectively, used the internet (The World Bank Group 2022), which results in very high levels of potential respondents. Despite targeting the internet public in general (dissemination of the questionnaires included wide-ranging social network groups), the audience reached may have some level of environmental awareness, i.e., have an above-average knowledge and awareness of environmental-related subjects such as invasive species. This may have occurred because online surveys require initiative from respondents, and citizens who are interested in environmental issues were probably more willing to answer the questionnaire (Fricker 2012), and also because survey dissemination also included social media of the LIFE Stop Cortaderia or INVASORAS.PT projects.

### Questionnaires and data collection

Besides the high potential outreach, an online questionnaire was chosen due to the speed of implementation, the difficulties of applying it by post or in-person simultaneously in the two countries, and due to the limitations during the Covid-19 pandemic. The questionnaire was pilot-tested with 10 people and their contributions were incorporated into the final version, consisting of a minor rephrasing of some questions. The questionnaire was made available online (using Google Forms) for approximately two months, from mid-April to mid-June 2020 and took about three minutes to complete. It was divided into two sections: Section I aimed to characterize the respondents and their basic knowledge about pampas grass; if the respondents did not recognize the species, the questionnaire finished in Section I; if they recognized it, the questionnaire continued to Section II, addressing more complex knowledge about the species. The questionnaire consisted of 11 questions, some with follow-up questions; most of the questions were close-ended, with only a few open-ended (Table 1; Supplementary Information 1, Table S1; Supplementary Information 2). The questionnaire was disseminated through different platforms, *e.g.,* wide-ranging social media groups, webpages and email lists from both the LIFE Stop Cortaderia and INVASORAS.PT projects. In each country, the questionnaire was distributed using their national platforms, but since the questionnaire was shared frequently through social media and emails, in practice, the target audience might have reached other countries.

**Table 1:** Summary of the questionnaire, including the type of questions

Section	Aim	Question	Type of question	
I	Characterization of the respondent	Q1, Q2, Q3, Q5	Close-ended	Multiple choice Single response
		Q4, Q5.1	Open-ended	Short answer
	Basic knowledge of the species	Q6, Q6.2	Close-ended	Multiple choice Single response
		Q6.1	Open-ended	Short answer
II	Complex knowledge of the species	Q7	Close-ended	Multiple choice Multiple response
		Q8, Q8.1, Q9		Multiple choice Single response
		Q8.2	Open-ended	Short answer
	Source of the knowledge	Q10	Close-ended	Multiple choice Multiple response
	Suggestions to plant instead of pampas grass	Q11	Open-ended	Long Answer/Paragraph

### Data analysis

Respondents' profile data were analysed using simple descriptive statistics. Answers to open-ended questions or with multiple choice were rearranged into coherent categories to facilitate further analysis (Supplementary Information 1, Table S2). Regarding respondents' occupations (Q4), the classification was based on Rodríguez-Rey et al. (2021). Occupations classified as the first sector (*e.g.,* farmers, fishermen, agronomists, landscape architects, …) were named henceforth as “related to nature”, since such professionals have direct contact with nature, namely species exploitation. This category is different from respondents categorized as environmental experts that include ecology researchers, biology teachers, natural resources managers, forest engineers, nature guides, etc.). Correctness of common names used in responses to identify *C. selloana* (Q6.1) followed Marchante et al. (2014) and González et al. (2020). Regarding Q7, the statements were classified as “most accurate” and “least accurate” by authors, following available knowledge, mostly from published sources; because some respondents choose both accurate and inaccurate statements, a third category was created to accommodate this. To evaluate if there was an association between the profile of the respondents (Section I, Q1 to Q5) and their knowledge and perception of pampas grass, a Chi-Square test (χ^2^) was applied between Q1 to Q5 and “basic knowledge of the plant” (Section I, Q6), “complex knowledge of the plant” (Section II, Q7 to Q9) and complementary information (Section II, Q10 and Q11). Meaningful associations (*e.g.*, the association between Q3—Education and Q6 – Do you recognize the plant?) are presented and discussed, while associations considered without a probable meaning were excluded (*e.g.*, the association between Q8—Is pampas grass an invasive plant in your country? and Q10—How did you know it is an invasive plant?). When assumptions of the Chi-Square test were not met, Fisher’s Exact test (FET) was used instead. In the case of an association between variables, the strength of that association was measured using the Phi for 2 × 2 tables and Cramer’s V for nxn tables; values below 0.2 refer to small associations, between 0.2 and 0.6 to medium associations and above 0.6 to large associations. An alpha level of 0.05 was used for all statistical tests. As the history of invasion and the date on which the species was listed as invasive in the legislation is different in the two countries, the analyses were performed on Portugal and Spain separately, using IBM SPSS® Statistics software, version 27.

## Results

1325 questionnaires were received: 486 from Portugal (37%) and 839 from Spain (63%). From these, 118 respondents (9%) did not recognize pampas grass, responding only to Section I; answers of both sections were analysed for the remaining 1207 (91%) (437 for Portugal and 770 for Spain).

### Profile of respondents

The majority of respondents live in the country where they answered the questionnaire (94.7% in Portugal—PT, 97.5% in Spain—ES), with a few exceptions of people who answered from other countries, *e.g.*, Germany, United Kingdom, Brazil, Italy and Switzerland (3%), among others. The majority of respondents were aged between 41 and 64, followed by 26 and 40, with just a few very young (under 18) and over 65 respondents taking the questionnaire. More women answered the questionnaire in PT (68.5 %), while in ES men and women were almost equally represented. Both in PT and ES, around 80% of respondents have completed higher education and about half work in trade and services (third sector) (43.4% in PT and 54.8% in ES), followed by environmental experts (22.8% in PT and 17.4% in ES) and only a small percentage had occupations related to nature (first sector) (Table 2).

**Table 2:** Profile of the respondents by country (Portugal—486 answers and Spain—839 answers). In Q4—Occupation, the first sector includes *e.g*., farmers, fishermen and miners, the second sector includes industrial managers, construction and factory workers, the third sector includes IT technicians, bank officers and mechanics, and environmental experts include biologists, biology teachers and forest engineers; “Other” refers to answers that were unperceived or too general to be included in any category, such as “dependent” or “autonomous”

		PT (*n* = 486) (%)	ES (*n* = 839) (%)
Q1. Age	< 18 years old	0.8	0.4
	18–25 years old	8.9	4.9
	26–40 years old	35.2	26.2
	41–64 years old	51.4	60.2
	65–89 years old	3.7	8.1
	> 90 years old	0.0	0.2
Q2. Gender	Woman	68.5	49.3
	Man	31.5	50.7
Q3. Education	Basic education	1.9	3.8
	High school	14.9	16.7
	Higher education	83.2	79.5
Q4. Occupation	First sector (related to nature)	6.2	1.5
	Second sector (producers/industry)	0.8	2.4
	Third sector (trade and services)	43.4	54.8
	Environmental experts	22.8	17.4
	Non-biology teachers	9.7	8.1
	Students	8.0	4.1
	Unemployed and retired	7.4	8.8
	Other	1.7	2.9
Q5. Country of residence	Portugal	94.7	0.1
	Spain	1.0	97.5
	Other	4.3	2.4

### Perception about *Cortaderia selloana*

The majority of respondents (89.9% in PT and 91.8% in ES) recognized pampas grass and, from these, 78.9% in PT and 89.4% in ES were able to name it correctly, using either the scientific or common name. Almost all respondents (99% and 98% in PT and ES, respectively), indicated that they do not have pampas grass on their property.

When the association between the profile of respondents and their knowledge was explored, medium and small associations were found between the Portuguese respondents' occupation (Q4) and whether they recognize pampas grass (Q6) (*p* = 0.003, FET; Cramer’s V = 0.214) and are able to name it (Q6.1) (*p* = 0.002, FET; Cramer’s V = 0.186); there was also a small association between age (Q1, *p* = 0.019, FET, Cramer’s V = 0.124) and knowing the name of pampas grass (Q6.1) (Supplementary Information 3, Table S1; for better visualisation, all statistical information of this section is detailed in Supplementary Information 3). All respondents with occupations related to nature (first sector), producers (second sector) and most students and environmental experts recognized the plant, while around 15% of respondents from the services sector (third sector), unemployed and retired people did not. As for the species name (Q6.1), around 80% of respondents knew it, but young people, adults and respondents with occupations related to nature, and environmental experts were more accurate (Supplementary Information 4, Fig. S1). Regarding Spanish respondents, there was a small association between age (Q1, *p* = 0.011, FET, Cramer’s V = 0.108) and gender (Q2, X^2^ (1, *N* = 827) = 5.171, *p* = 0.023, Cramer’s V = 0.079) and recognizing the species (Q6). There was also a small association between gender (Q2, X^2^ (3, *N* = 758) = 13.885, *p* = 0.003, Cramer’s V = 0.135) and knowing the name of pampas grass (Q6.1) (Supplementary Information 3, Table S1). Adult women recognized the plant more often, while more men attributed an incorrect name (Supplementary Information 4, Fig. S2).

**Fig. 1:**
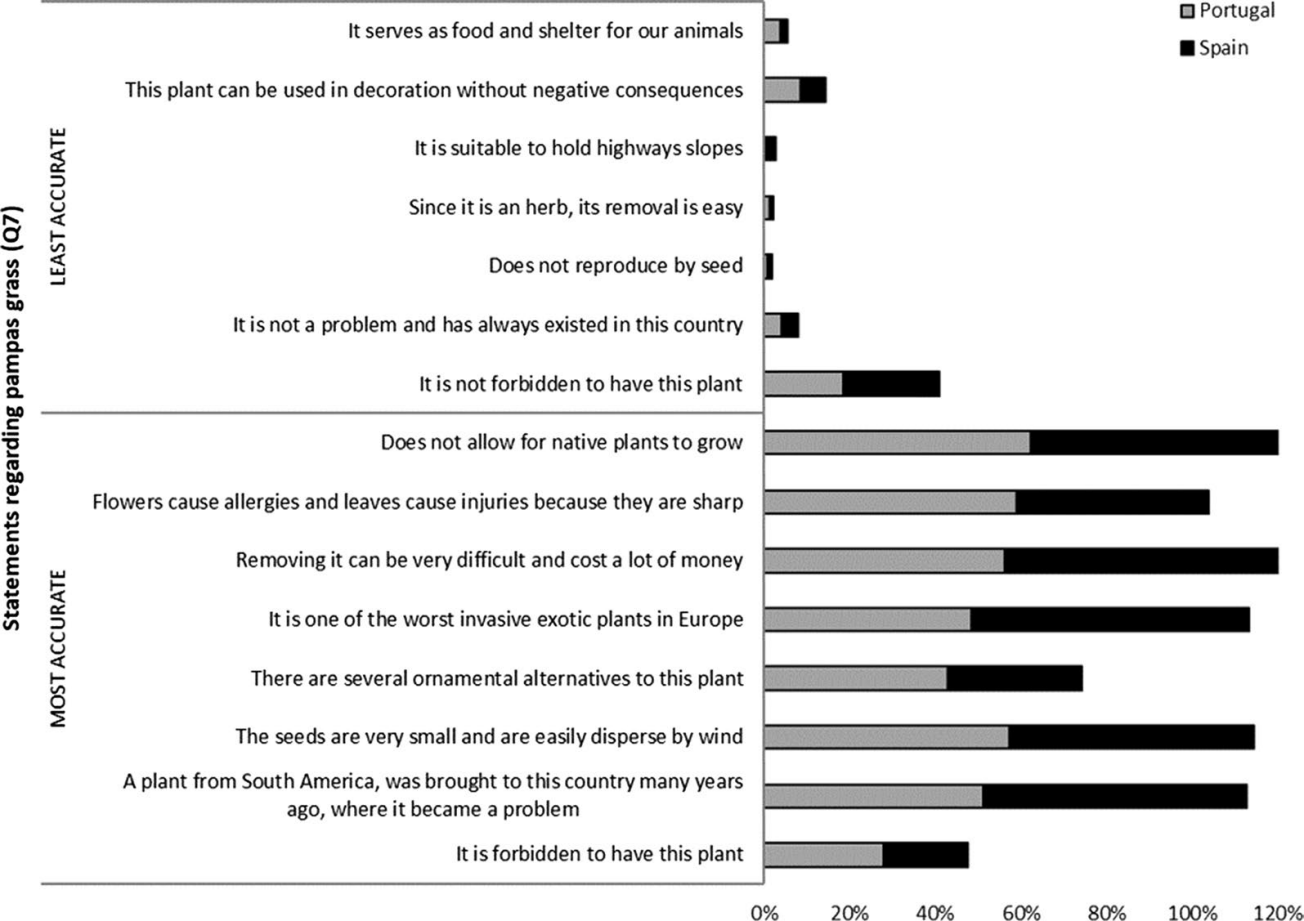
Answers from Portuguese and Spanish respondents to Question 7—“Select the statements that, in your opinion, are most appropriate for this plant”, *n* = 1207. The original categories were classified as “most accurate” and “least accurate” for further analysis

When presented with different statements about pampas grass (Q7), two-thirds of respondents selected only most accurate statements (71.6% in PT and 66.8% in ES), *e.g.,* “does not allow for native plants to grow” or “removing it can be very difficult and cost a lot of money”; a small percentage (6.6% in PT and 5.3% in ES) selected only least accurate answers, *e.g.,* “it is not forbidden to have this plant” and “this plant can be used in decoration without negative consequences”; and 21.7% in PT and 27.9% in ES selected both accurate and inaccurate statements (Fig. 1).

In Portugal, respondents’ occupation (Q4; *p* = 0.002, FET; Cramer’s V = 0.197) showed a small association with the choice of the statements best suited for pampas grass (Q7), while in Spain, it was respondents' age (Q1; *p* = 0.000, FET; Cramer’s V = 0.112) and gender (Q2; *p* = 0.002, FET; Cramer’s V = 0.130) that presented a small association with Q7 (Supplementary Information 3, Table S2). Portuguese producers had the highest percentage of most accurate statements, followed by environmental experts and related to nature occupations; on the other hand, non-biology teachers and the services sector respondents selected most of the least accurate statements. Spanish young and adult women selected more accurate statements than any of the other groups (Supplementary Information 4, Fig. S3).

**Fig. 2:**
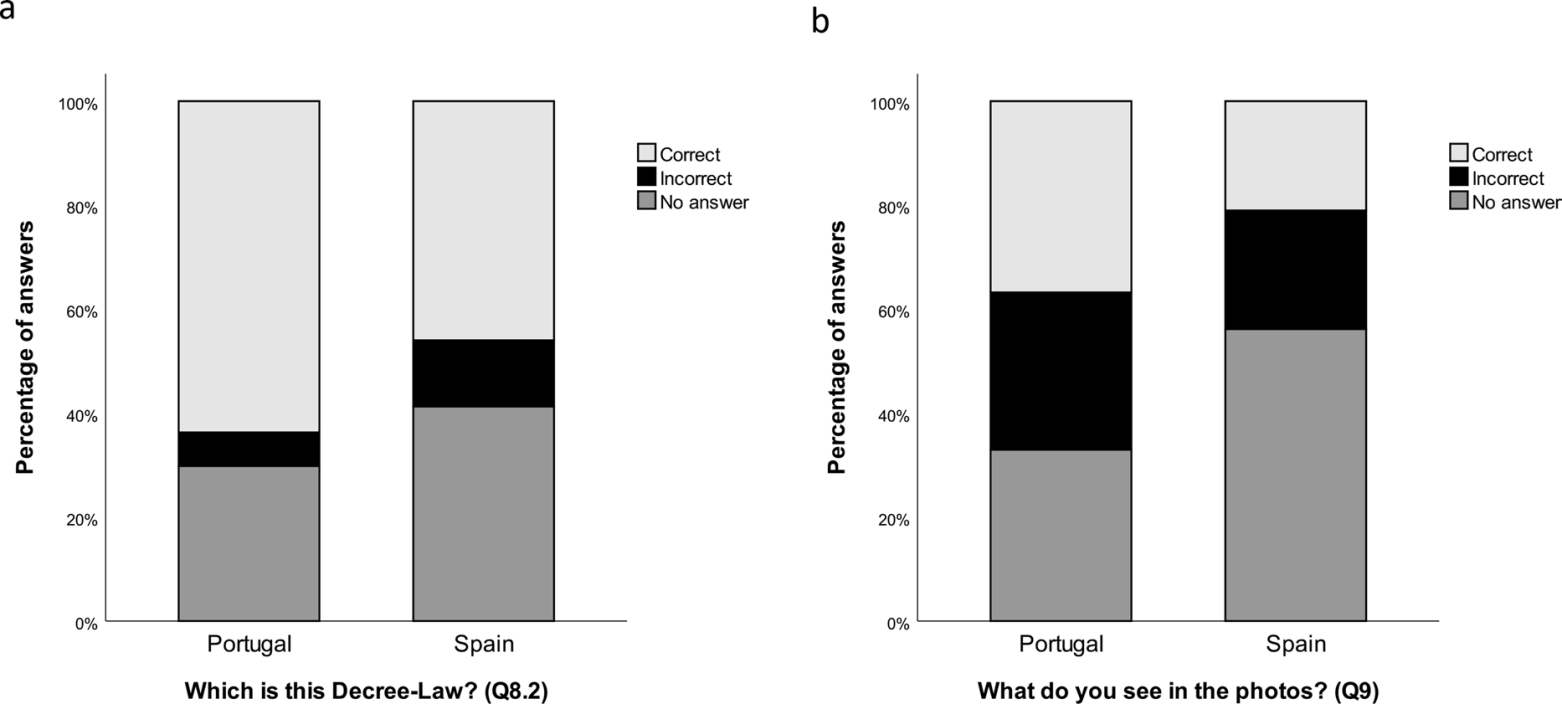
Answers from Portuguese and Spanish respondents to the questions Q8.2—“If you answered yes to the previous question, which is this Decree-Law?”, *n* = 250 **a** and Q9—“In your opinion, what do you see in the photos below?”, *n* = 1027 **b** The original categories were classified as “correct”, “incorrect” and “no answer” for further analysis

When asked if pampas grass is invasive (Q8) and included or not in any legislation (Q8.1, Q8.2), most respondents recognized that the species is invasive (90.4% in PT and 92.6% in ES) but many were not aware that a specific legislation limits the species’ use (68.8% in PT and 82.9% in ES). Nevertheless, a big part of the respondents (63.7% in PT and 46.0% in ES; Fig. 2a) who knew about the legislation also knew the correct name/number of the legal document, *i.e.,* Decree-Law nº 92/2019 in Portugal and Royal Decree nº 630/2013 in Spain, despite this being more pronounced in Portugal.

Respondents’ occupation (Q4) was  moderately associated with the recognition that pampas grass is invasive (Q8) for Portugal (*p* = 0.001, FET; Cramer’s V = 0.232) and little associated for Spain (*p* = 0.011, FET; Cramer’s V = 0.168); in Spain, there was also a medium association with respondents' age (Q1; *p* = 0.000, FET; Cramer’s V = 0.227) (Supplementary Information 3, Table S3). In Portugal, all respondents from the second sector and those with occupations related to nature recognized the invasive behaviour of pampas grass, followed by environmental experts and unemployed and retired people, while almost 20% of respondents working in the service sector did not recognize the species as invasive. Regarding Spain, most young and adult people and all respondents with related to nature occupations replied that pampas grass was invasive, followed by environmental experts, but more than 25% of students said the species was not invasive (Supplementary Information 4, Fig. S4).

Respondents’ education (Q3) showed a small association with awareness of legislation that limits pampas grass in both countries (Q8.1; Portugal: X^2^ (2, *N* = 392) = 12.513, *p* = 0.002, Cramer’s V = 0.179 and Spain: X^2^ (2, *N* = 707) = 12.124, *p* = 0.002, Cramer’s V = 0.131); occupation (Q4) also showed a medium association with Q8.1, but for Portugal only (*p* < 0.001, FET; Cramer’s V = 0.286) (Supplementary Information 3, Table S3). Respondents with higher education in both countries, and environmental experts and occupations related to nature in Portugal, acknowledged the existing legislation better (Supplementary Information 4, Fig. S5).

Regarding the identification of legislation (Q8.2), there was a medium association with respondents’ occupation (Q4) in Portugal (*p* = 0.008, FET; Cramer’s V = 0.328) and with respondents’ education (Q3) in Spain (*p* = 0.014, FET; Cramer’s V = 0.200) (Supplementary Information 3, Table S3). More Portuguese producers and unemployed and retired people were aware of the specific Decree Law, while in Spain, respondents with higher education gave the most correct answers (Supplementary Information 4, Fig. S6).

When three detailed photos of pampas grass were presented, asking what the respondents saw (Q9), a high percentage of respondents did not reply/ knew the answer (PT 33.0%, ES 56.1%); in each country, the percentage of people who gave correct and incorrect answers was similar (PT: 36.8% correct and 30.2% incorrect; ES: 21.0% correct and 22.9% incorrect) (Fig. 2b). Portuguese respondents’ education (Q3; *p* = 0.000, FET; Cramer’s V = 0.199) and occupation (Q4; *p* = 0.003, FET; Cramer’s V = 0.188) showed a small association with identifying correctly the pampas grass photos (Q9) (Supplementary Information 3, Table S4): unemployed and retired people, environmental experts and respondent’s with higher education gave the most correct answers, while producers and respondents with basic education got it wrong more often (Supplementary Information 4, Fig. S7).

### Complementary information

**Fig. 3:**
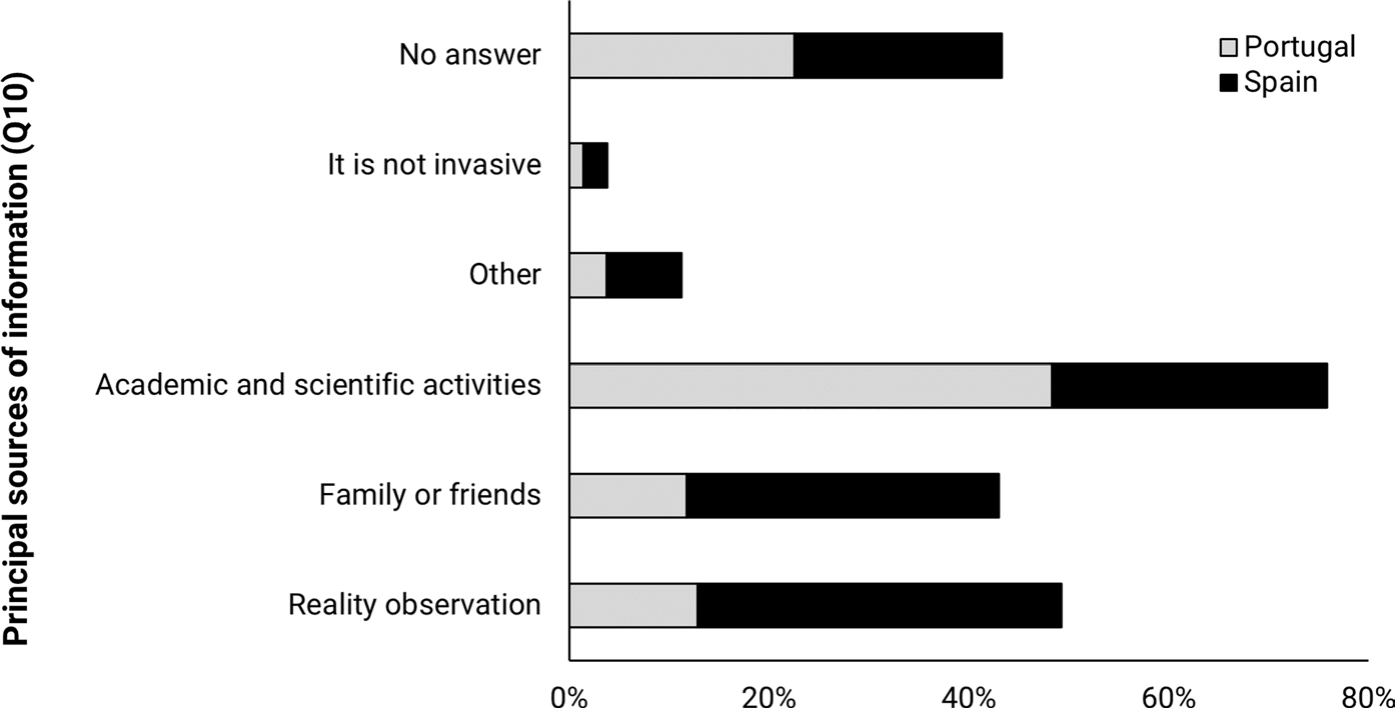
Main sources from which respondents learned that pampas grass is an invasive species (Q10), *n* = 1207

**Fig. 4:**
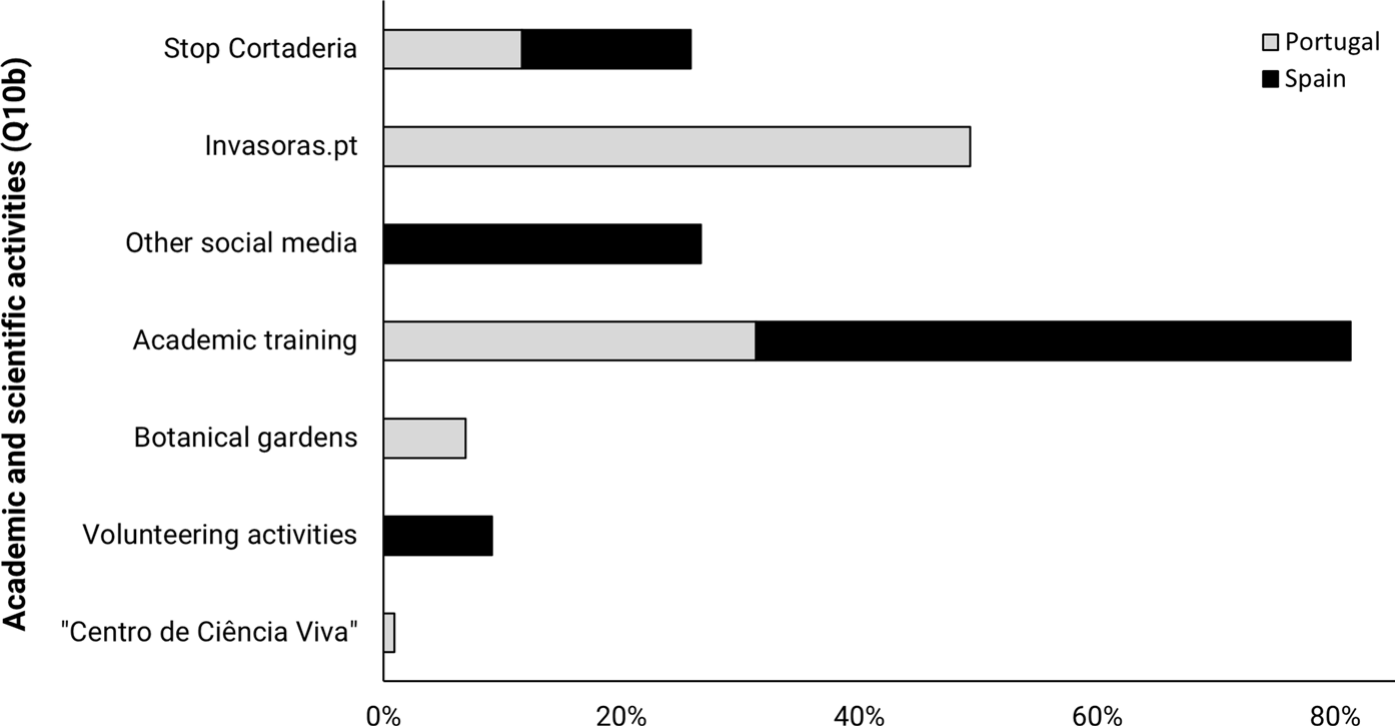
Academic and scientific activities (Q10b) from which respondents learned that pampas grass is an invasive species, *n* = 353

More than half of Portuguese respondents learned that pampas grass is invasive (Q10) through academic and scientific activities (which grouped several options from the list given: Supplementary Information 1, Table S2), while for the Spanish respondents the observation of reality and family or friends were the main sources of information (Fig. 3). When considering academic and scientific activities separately (Q10b), academic training was the main source of information for acknowledging pampas grass as an invasive species in Spain, whereas in Portugal it was the platform INVASORAS.PT (including webpage, social media and activities) (Fig. 4). No meaningful association was found between respondents' profiles and the main sources they learned that pampas grass is invasive (Q10).

**Fig. 5:**
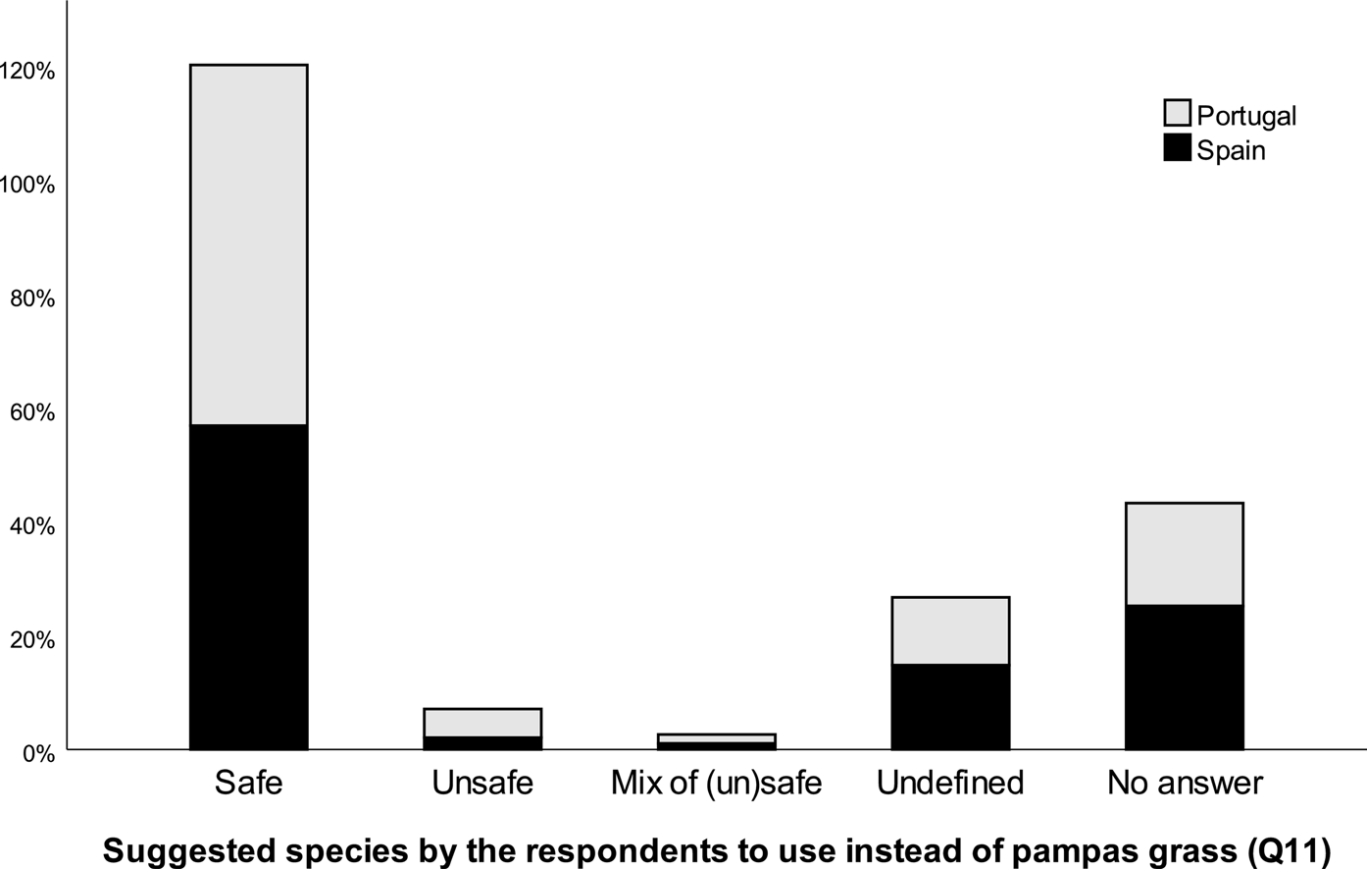
Categories of species suggested by the respondents to use as ornamentals in gardens instead of pampas grass (Q11), *n* = 1207. The categories are as follows: Safe (both native and non-invasive exotic species), Unsafe (invasive and exotic species with invasive potential in limited situations), Mix of (un)safe (when both safe and unsafe plants were suggested by the same respondent) and Undefined (when generalist names were used, which made it impossible to categorize the species)

Finally, when asked about alternative species to use in gardens instead of pampas grass (Q11), most respondents, in both countries, suggested “safe” plants (i.e., native species and/or non-invasive exotic species), although a reasonable proportion was not able to provide an alternative (Fig. 5). A minority suggested “unsafe” plants (i.e., invasive or potentially invasive species) as alternatives, a few of them being invasive, such as *Acacia dealbata* Link, *Arundo donax* L. and *Carpobrotus edulis* (L.) N. E. Br..

There was only a small association between respondents' occupation (Q4) and alternative species suggested to use instead of pampas grass (Q11) in Portugal, but it was non-significant (*p* = *0.060*).

## Discussion

Despite the undeniable invasiveness, and public awareness campaigns, citizens still grow in their gardens and/or collect pampas grass flowers for decorative use and, in doing so, they may disperse the seeds (if available), so it is particularly important to understand people’s perception of *Cortaderia selloana*. Our results show that a very high percentage of Portuguese and Spanish respondents not only recognize and identify pampas grass but also acknowledge its invasive behaviour. This high level of knowledge and perception may reflect some bias in the audience reached (see below) but is in line with previous studies with other invasive alien plants widely dispersed that are also recognized, as well as their invasive status, by a large proportion of citizens (Dehnen-Schumutz et al. 2010; Junge et al. 2019; Cordeiro et al. 2020). Citizens recognized pampas grass more (91% in both countries in this study) than other well-known established IAS such as the silver wattle (*Acacia dealbata*; 69% in Cordeiro et al. 2020) and other *Acacia* species (70% for *A. dealbata* and lower for other *Acacia* species in Vaz et al. 2019). These species are older introductions and more widespread, but pampas grass has increased its distribution faster and more notably in the last two decades, is more commonly used for decoration and is a very showy species (Marchante et al. 2014; González et al. 2020), which help to explain these results. In this regard, it may be interesting to include in future questionnaires the perception regarding the positive–negative impacts (*e.g.*, costs-benefits, ecosystem services-disservices) of the species, instead of focusing only on the negative ones (Vaz et al. 2017; Sax et al. 2022).

The general knowledge about pampas grass and its invasive status was found to be equivalent in Portugal and Spain. Since pampas grass is a very showy and decorative species, easy to identify, and has been widely planted around the Iberian Peninsula as an ornamental plant in public and private gardens (González et al. 2020), its recognition through a single photograph was expected, even if respondents do not have the plant in their properties. However, a surprisingly high percentage of respondents recognized its invasive status, contrasting with everyday experiences. In fact, people use pampas grass plumes decoratively in shops and restaurants, and social media *influencers* sometimes recommend their use in social events (*e.g.,* on Instagram). Additionally, in public awareness activities, participants frequently say that pampas grass is beautiful and admit not being aware of its invasiveness (*authors'* personal communication). These results can be possibly explained by our respondents’ above-average level of environmental awareness. This bias is somewhat common. As Fricker R.D. (2012) underlines, since online surveys require the initiative of respondents, citizens interested in environmental issues are likely to be more willing to answer questionnaires environmentally related. On the other hand, these results may also reflect the success of the large investment of LIFE Stop Cortaderia over the last three years in both countries (González et al. 2020; Association Amica 2021) and INVASORAS.PT platform for almost two decades in Portugal (Marchante and Marchante 2016; Marchante et al. 2017; Cordeiro et al. 2020), to raise awareness of the pampas grass. Still, despite recognizing pampas grass and its invasive potential, only a small proportion of respondents, mainly those with higher education, was aware of the existence of legislation that limits its use.

Some associations were found between respondents’ demographic characteristics and their perception and knowledge of pampas grass, most often age, education and occupation: in Portugal, associations were stronger with respondents' occupation, while in Spain they were stronger with respondents’ age and education. Young (underrepresented) and adults with higher education, occupations related to nature (first sector), environmental experts (low-represented) and producers (second sector), were, in general, associated with higher percentages of correct answers or more knowledge about pampas grass. This suggests that higher education and formal training in environmental areas influence perception and increase knowledge about invasive species, as found by other authors (White et al. 2005; Lindemann-Matthies 2016; Potgieter et al. 2019; Cordeiro et al. 2020). In fact, academic and scientific activities, possibly most sought after by people with higher education, and particularly academic training and the platform INVASORAS.PT in Portugal, were selected as the main source for respondents learning about the invasive behaviour of pampas grass. This corroborates previous studies showing that informal education, along with formal education, increases knowledge and alters perceptions about invasive species (Bremner and Park 2007; Schreck Reis et al. 2013; Cordeiro et al. 2020; Sosa et al. 2021). In Spain, real-world observation and conversations with family and friends seemed to also play a major role in respondents’ knowledge about pampas grass. This might be explained by the fact that the invasion by pampas grass is far more widespread, with more extensive continuous areas occupied by the species, in the north of Spain than in Portugal, specifically in Cantabria (González et al. 2020). Indeed, many Spanish respondents possibly originate from this region, as LIFE Stop Cortaderia is based in Cantabria  and has many followers on social media. In addition, the invasion by pampas grass in Spain is older, with governmental and even public health entities acknowledging the problem more (Herrera and Campos 2006; MAGRAMA 2013; Gobierno de Cantabria 2017; Gomez et al. 2018; Rodríguez et al. 2021) than in Portugal, where the species was included in the legislation as invasive species only in 2019 (Ministério do Ambiente 2019). In areas where the species is widely dispersed, such as the northern coast of the Iberian Peninsula, many citizens already recognize its invasive status and negative consequences, and this increased awareness may represent a growing willingness to halt the expansion of pampas grass and even collaborate on strategies for its control, removal and even availability to answer this questionnaire. Increased public awareness is a key factor for the success of invasive species management, especially in areas where the species occurs more sporadically and citizens' participation is essential to locate it, remove it and achieve local eradication. In this context, the strong impact of projects such as LIFE Stop Cortaderia, in both countries, and INVASORAS.PT in Portugal, should not be discounted. Both projects have a strong investment in raising social and stakeholders’ awareness regarding the harmful effects of invasive plants, in general, and pampas grass in particular, in the natural and transformed ecosystems (Marchante and Marchante 2016).

Despite the general high level of awareness shown by the results, between 15 and 20% of respondents from the third sector (trade and services) (particularly in Portugal, where they were over 40% of all respondents) either did not recognize the species or its invasive status or chose inaccurate statements to describe it (Supplementary Information 4). This particular group shows a lack of awareness of this invasive species, considering pampas grass a beautiful ornamental plant that does not pose a threat, a common situation we encounter during awareness-raising activities. This is a general conception for many appealing invasive species (Potgieter et al. 2019), highlighting the need to focus more awareness-raising activities on such target audiences.

Some biases were identified that prevented us from sampling a more representative subgroup of the entire Iberian population. The respondents were probably more interested in environmental issues than the average citizen (see discussion above). They were mostly adults, probably because this theme is not of interest to the younger public, many disconnected from nature (Battisti 2016; Battisti et al. 2018). In addition, being distributed exclusively online, the questionnaire was eventually less reachable by senior citizens (Rebelo 2013). These biases could have been overcome with face-to-face questionnaires, but such an approach would have probably limited the number of responses. Online surveys allow for reaching more people from different geographic regions, with less effort and costs, since no fieldwork is necessary, thus facilitating international studies. They also allow to maintain anonymity, which can eliminate some interviewer biases and intimidation caused by the surveyor, allowing the respondents to express themselves more genuinely (Bird 2009; Pozzo et al. 2019).

Considering the above-mentioned biases, our results cannot be generalized to the entire Iberian population, but represent an interesting segment of the population and are certainly valid and a good example of what can be achieved with public education and outreach campaigns. Equivalent results were shown by Oele and collaborators (2015), where a significant decrease in the selling of aquatic invasive species was observed after education efforts.

## Conclusions

Our study suggests a reasonably high level of knowledge and perception from environmentally aware Portuguese and Spanish citizens that use the internet regarding the invasive pampas grass (*Cortaderia selloana*). This target public recognizes the species and its invasive behaviour and is able to name it, but is less knowledgeable about the legislation that limits its use. The results from Portuguese and Spanish respondents were relatively similar, despite occupation being the factor that in Portugal showed more association with the respondents' knowledge and perception, while in Spain it was age and education. In general, citizens with occupations related to the services sector, environment and nature showed more knowledge, along with citizens with higher education. Although results cannot be extrapolated to the entire Iberian population, it highlights the importance of investing in public awareness and education campaigns, such as those developed by LIFE Stop Cortaderia and INVASORAS.PT. These campaigns are essential to change citizens' perception and knowledge about an attractive ornamental species, but with serious consequences to the environment, public health and the economy.

## Supplementary Information

Below is the link to the electronic supplementary material.

Supplementary file 1 (PDF 118 KB)

Supplementary file 2 (PDF 417 KB)

Supplementary file 3 (PDF 129 KB)

Supplementary file 4 (PDF 684 KB)

## Data Availability

Authors declare that the records of the original data are available.
