# Supplementary material for: Public perceptions about the invasive pampas grass, Cortaderia selloana: a case study of environmentally conscious citizens in Southern Europe
Source: Biol Invasions. 2023 Mar 28;25(6):2043–56. doi: 10.1007/s10530-023-03025-3 (PMC10042667; doi:10.1007/s10530-023-03025-3)
Supplement: Supplementary file 1 — (PDF 118 KB) [file 10530_2023_3025_MOESM1_ESM.pdf]

Questions available in the questionnaire and their reclassification for further analysis

Table S1 Questions available in the questionnaires translated to English (the originals were in Portuguese and Spanish)

Question number	Question text
**Q1**	Age
**Q2**	Gender
**Q3**	Education
**Q4**	Occupation
**Q5**	Country of residence (Portugal, Spain)
**Q5.1**	Country of residence (another country)
**Q6**	Do you recognize the plant in the photo?
**Q6.1**	If yes, what´s the name of the plant?
**Q6.2**	Do you have this plant on your property?
**Q7**	Select the statements that, in your opinion, are most appropriate for this plant.
**Q8**	Is pampas grass an invasive plant in your country?
**Q8.1**	If you answered yes to the previous question, do you know of any Decree-Law that limits its use?
**Q8.2**	If you answered yes to the previous question, which is this Decree-Law?
**Q9**	In your opinion, what do you see in the photos below?
**Q10**	How did you find out that pampas grass is an invasive plant?
**Q11**	Finally, and as a challenge, instead of the pampas grass, what other plant(s) would you use as ornamentals in gardens?

Table S2 Data reclassification for further analysis. Only those which were reclassified are shown.

Question number	Categories	Reclassification
**Q1. Age***	< 18 years old	Young
	18-25 years old	Adult
	26-40 years old	Adult
	41-64 years old	Adult
	65-89 years old	Old
	> 90 years old	Old
**Q4. Occupation**	*Open-ended question*<br><br>*Classification based on* Rodríguez-Rey et al. (2021)	First sector (primary production, related to nature; *e.g.* farmers, fishermen …)<br>Second sector (producers / industry)<br>Third sector (trade and services; *e.g.* pharmaceuticals, lawyers, managers, administratives …)<br>Environmental experts (e.g. ecology researchers, biology teachers, natural resources managers, forest engineers, nature guides …)<br>Students<br>Non-biology teachers<br>Unemployed and retired people
**Q6.1 If yes, what´s the name of the plant?**	*Open-ended question*	Correct name<br>Incorrect name<br>No answer
**Q7. Select the statements that, in your opinion, are most appropriate for this plant.**	It serves as food and shelter for our animals	Least accurate statements
	This plant can be used in decoration without negative consequences	Least accurate statements
	It is suitable to hold highways slopes	Least accurate statements
	Since it is an herb, its removal is easy	Least accurate statements
	Does not reproduce by seed	Least accurate statements
	It is not a problem and has always existed in this country	Least accurate statements
	It is not forbidden to have this plant	Least accurate statements
	Does not allow for native plants to grow	Most accurate statements
	Flowers cause allergies and leaves cause injuries because they are sharp	Most accurate statements
	Removing it can be very difficult and cost a lot of money	Most accurate statements
	It is one of the worst invasive exotic plants in Europe	Most accurate statements
	There are several ornamental alternatives to this plant	Most accurate statements
	The seeds are very small and are easily dispersed by wind	Most accurate statements
	A plant from South America, was brought to this country many years ago, where it became a problem	Most accurate statements
	It is forbidden to have this plant	Most accurate statements
**Q8.2 If you answered yes to the previous question, which is this Decree-Law?**	*Open-ended question*	Correct<br>Incorrect<br>No answer

**Q9. In your opinion, what can be seen in the photos below?**	Flowers of three different species of grasses	Incorrect
	The first two belong to *C. selloana* and the last to *C. jubata*	Correct
	All photos are *C. selloana*, different sexes	
	Doesn´t know / No reply	No answer
**Q10. How did you know that pampas grass is an invasive plant?**	Reality observation	Reality observation
	Family or friends	Family or friends
	Stop Cortaderia (webpage, social media, training)<br>Invasoras.pt (webpage, social media, activities)<br>Other social media<br>Academic training<br>Botanical gardens<br>Centro de Ciência Viva	Academic and scientific activities
	It is not invasive	It is not invasive
	Other (*e.g.* this questionnaire, professional activity, CTC, CCV, traditional media, legislation, self-taught)	Other
	Don´t remember<br>No answer	No answer
**Q11. Finally, and as a challenge, instead of the pampas grass, what other plant(s) would you use as ornamentals in gardens?**	*Open-ended question*	Safe (both native and exotic non-invasive species)
		Unsafe (invasive and exotic species with invasive potential in limited situations)
		Mix of (un)safe (when both safe and unsafe plants were suggested by the same respondent)
		Undefined (when generalist names were used, which made it impossible to categorize the species)
		No answer

* grouped only when analyzing the association between questions.
