# Supplementary material for: Public perceptions about the invasive pampas grass, Cortaderia selloana: a case study of environmentally conscious citizens in Southern Europe
Source: Biol Invasions. 2023 Mar 28;25(6):2043–56. doi: 10.1007/s10530-023-03025-3 (PMC10042667; doi:10.1007/s10530-023-03025-3)
Supplement: Supplementary file 2 — (PDF 417 KB) [file 10530_2023_3025_MOESM2_ESM.pdf]

QUEM É A ERVA-DAS-PAMPAS?

Este inquérito é realizado no âmbito do projeto "LIFE Stop Cortaderia - Medidas urgentes para controlar a propagação de erva-das-pampas (Cortaderia selloana) no Arco Atlântico" que tem como principal objetivo a implementação de uma estratégia transnacional comum para lutar contra a erva-das-pampas ao longo do Arco Atlântico, de Portugal até França, passando por toda a costa Cantábrica.

O inquérito é anónimo, pelo que pedimos total sinceridade nas respostas. Demora cerca de 3 minutos a responder. Os resultados serão depois publicados na página do projeto (stopcortaderia.org) e no facebook (#stopcortaderia). Obrigado :)

*Required

1. Idade *

- <18 anos
- 18-25 anos
- 26-40 anos
- 41-64 anos
- 65-89 anos
- >90 anos

2. Sexo *

- Feminino
- Masculino
- Prefiro não dizer

3. Escolaridade Completa *

- Ensino Básico
- Ensino Secundário
- Ensino Superior
- *Other:*

4. Profissão * ____

5. País de residência *

- Portugal
- Espanha
- França
- Outro

5.1 Se respondeu "Outro" à pergunta anterior, em que país reside? ______________________________

6. Reconhece a planta da seguinte fotografia? *

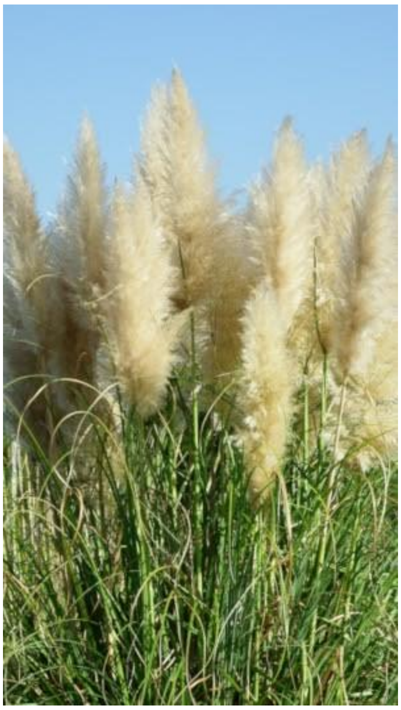

- Sim
- Não

6.1. Se respondeu sim à pergunta anterior, como lhe chama? ______________________________

6.2. Tem esta planta no seu jardim ou terreno?

- Sim
- Não

7. Selecione as afirmações que, na sua opinião, mais se adequam a esta planta *

- Serve de alimento e abrigo para os nossos animais
- Não deixa as plantas nativas (que sempre existiram em Portugal) desenvolverem-se
- É uma planta que pode ser utilizada em decoração porque não tem qualquer consequência negativa para o ambiente
- As flores provocam alergias e as folhas ferimentos por serem cortantes
- É a planta certa para segurar os taludes das autoestradas
- A sua remoção é fácil, pois trata-se de uma erva
- A sua remoção pode ser muito difícil e custar muito dinheiro
- É uma das piores plantas exóticas invasoras na Europa
- Existem várias alternativas ornamentais a esta planta
- As sementes são muito pequenas e dispersam-se facilmente com o vento
- A erva-das-pampas não se reproduz por semente
- Esta planta não é um problema em Portugal e sempre existiu por cá
- Esta planta é originária da América-do-Sul e foi trazida para Portugal há muitos anos, tendo-se tornado um problema nas últimas décadas
- É proibido ter esta planta em Portugal
- Não é proibido ter esta planta em Portugal

8. A erva-das-pampas é uma planta invasora em Portugal? *

- Sim
- Não

8.1. Se respondeu sim à pergunta anterior, tem conhecimento de algum Decreto-Lei que limite a sua utilização?

- Sim
- Não

8.2. Se respondeu sim à pergunta anterior, qual é esse Decreto-Lei? ______________________

9. Na sua opinião, o que se observa nas fotos abaixo? *

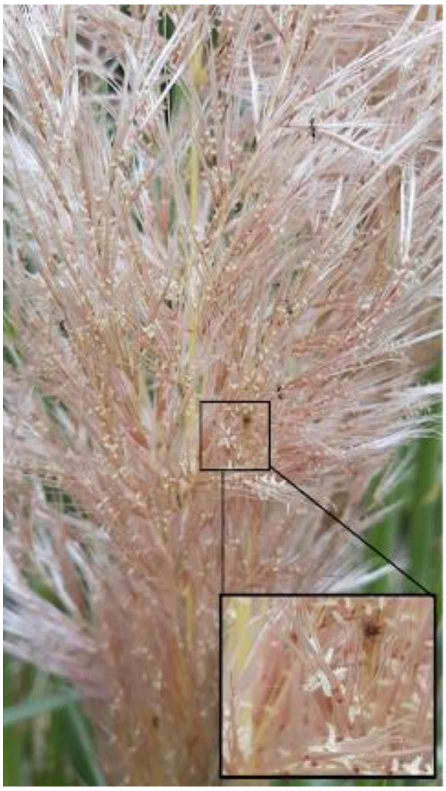
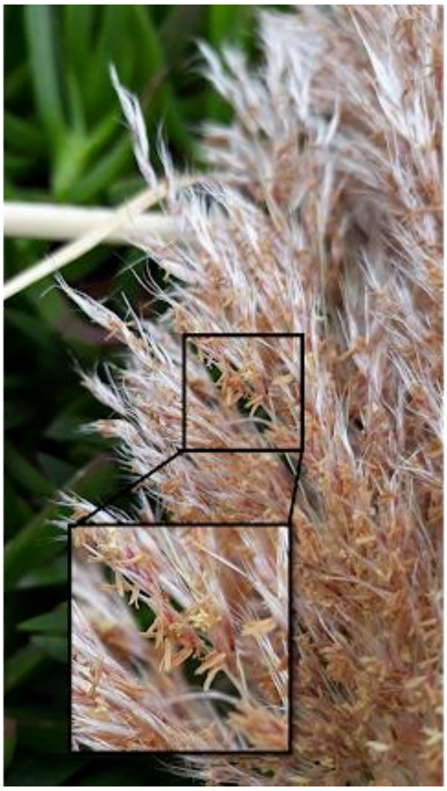
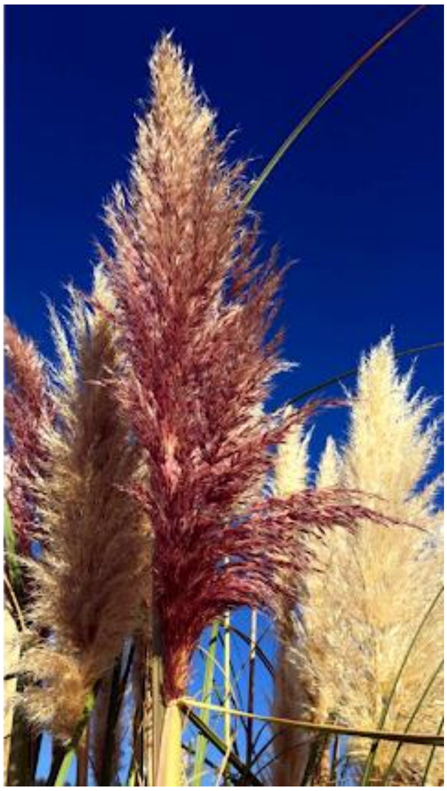

- Flores de três espécies diferentes de gramíneas
- As duas primeiras fotos são de Cortaderia selloana, enquanto que a última é de Cortaderia jubata
- Todas as fotos são de Cortaderia selloana, mas na primeira está uma pluma feminina e na segunda uma pluma hermafrodita
- Não sei

10. Como teve conhecimento de que esta espécie é invasora? *

- Observação da realidade
- Através do site Stop Cortaderia
- Através das redes sociais Stop Cortaderia
- Numa formação LIFE Stop Cortaderia
- Através do site Invasoras.pt
- Através do facebook "Espécies Invasoras em Portugal"
- Através de uma atividade da equipa "Espécies Invasoras em Portugal"
- Durante formação académica
- Através de familiares ou amigos
- Através de informação num jardim botânico ou páginas de internet relativas a jardins botânicos
- Não me lembro
- A erva-das-pampas não é invasora
- Other: ____

11. Para terminar e em jeito de desafio, em vez da erva-das-pampas, que outra(s) planta(s) utilizaria como ornamental em jardins? *

____

Obrigado pela sua participação!

Para saber mais sobre esta espécie e a sua problemática
LIFE Stop Cortaderia:
stopcortaderia.org
facebook, instagram, twitter:
#stopcortaderia
stopcortaderiapt@gmail.com

Invasoras.pt:
http://invasoras.pt/gallery
/cortaderia-selloana/
facebook, instagram,
twitter: #invasoraspt
invader@uc.pt

This content is neither created nor endorsed by Google.

Google Forms

QUIÉN ES EL PLUMERO?

Esta encuesta se realiza en el ámbito del proyecto "LIFE Stop Cortaderia - Medidas urgentes para el control de la expansión del plumero de la Pampa (Cortaderia selloana) en el Arco Atlántico" que tiene como principal objetivo la implementación de una estrategia transnacional común para luchar contra el plumero a lo largo del Arco Atlantico, de Portugal hasta Francia, pasando por toda la costa Cantábrica.

La encuesta es anónima, por lo que pedimos total sinceridad en las respuestas. Lleva cerca de 2 minutos responder. Los resultados serán después publicados en la página del proyecto (stopcortaderia.org) y en facebook (#stopcortaderia). Gracias **Required*

1. Idade *

- <18 años
- 18-25 años
- 26-40 años
- 41-64 años
- 65-89 años
- >90 años

2. Sexo *

- Femenino
- Masculino
- Prefiero no decirlo
-

3. Estudios Completados *

- Educación básica
- Educación secundaria
- Educación superior
- *Other:*

4. Profesión * ____

5. País de residencia *

- España
- Portugal
- Francia
- Otro

5.1 Si respondió "Otro" en la pregunta anterior, ¿en qué país reside? ____________________

6. ¿Reconoce la planta de la siguiente fotografía?

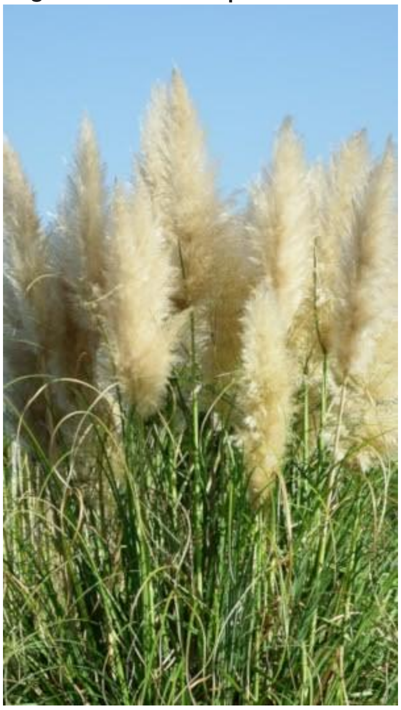

- Sí
- No

6.1. Si respondió sí a la pregunta anterior, ¿cómo la llama? ________________________________

6.2. ¿Tiene esta planta en su jardín?

- Sí
- No

7. Seleccione las afirmaciones que, en su opinión, más se adecuan a esta planta *

- Sirve de alimento y abrigo a nuestros animales
- No deja desarrollarse a las plantas autóctonas (las que siempre han existido en España)
- Es una planta que se puede utilizar en decoración porque no tiene consecuencias negativas para el medio ambiente
- Las flores causan alergias y las hojas heridas porque son cortantes
- Es una planta adecuada para asegurar los taludes de las autopistas
- Su eliminación es fácil, dado que se trata de una hierba
- Su eliminación puede ser muy difícil y costar mucho dinero
- Es una de las peores plantas invasoras de Europa
- Existen varias alternativas ornamentales a esta planta
- Sus semillas son muy pequeñas y se dispersan fácilmente con el viento
- El plumero no se reproduce por semillas
- Esta planta no es un problema en España y siempre existió por aqui
- Esta planta es originaria de América del Sur y fue traída a España hace muchos años y se ha convertido en un problema en las últimas décadas
- Está prohibido tener esta planta en España

No está prohibido tener esta planta en España

8. ¿El plumero es una planta invasora en España? *

- Sí
- No

8.1. Si respondió sí a la pregunta anterior, ¿tiene conocimiento de alguna ley que limite su utilización?

- Sí
- No

8.2. Si respondió sí a la pregunta anterior, ¿cuál es esa ley?_______________________

9. En su opinión, ¿ qué se observa en las siguientes fotos?

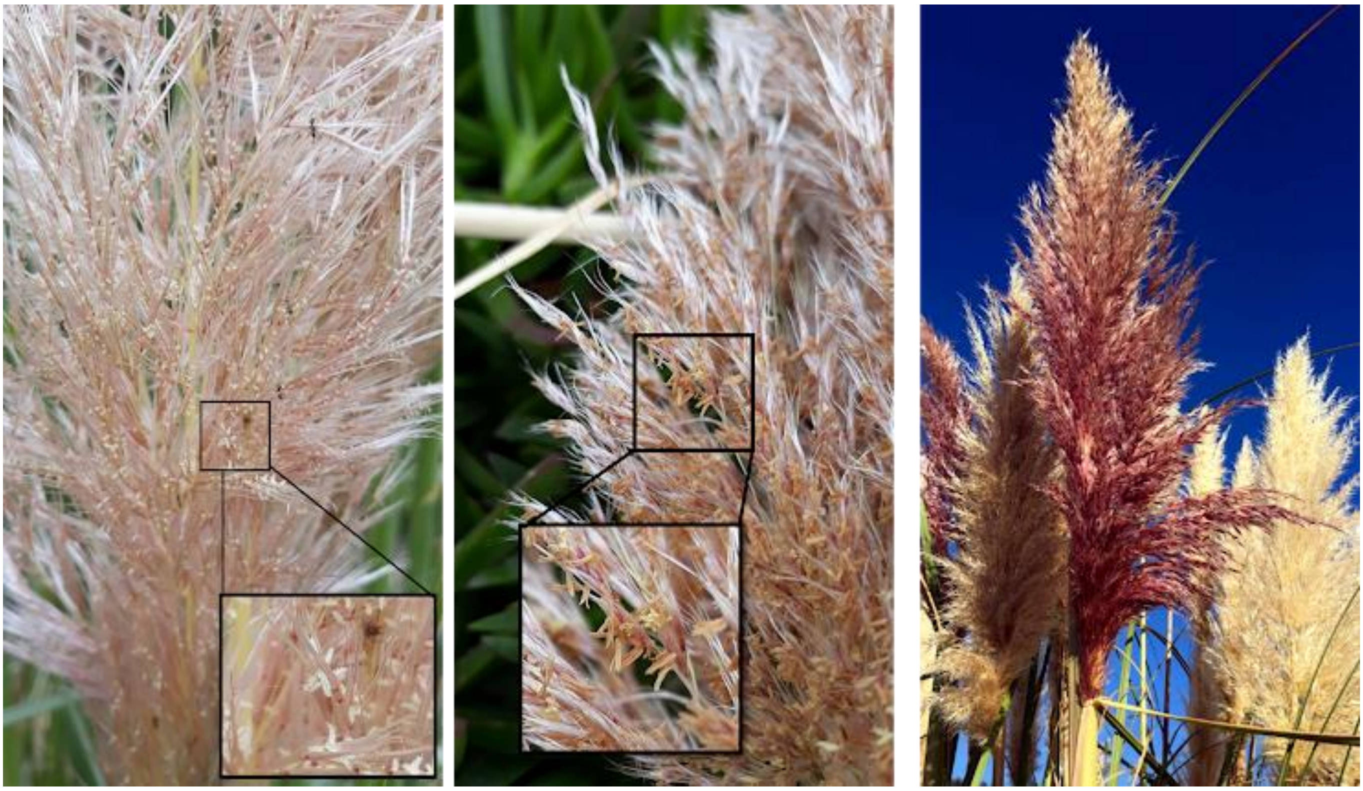

- Flores de tres especies diferentes de gramíneas
- Las dos primeras fotos son de Cortaderia selloana, mientras que la última es de Cortaderia jubata
- Todas las fotos son de Cortaderia selloana, pero la primera es una pluma femenina y la segunda una pluma hermafrodita
- No sé

10. ¿Cómo tuvo conocimiento de que esta especie es invasora? *

- Observando la realidade
- A través de la web de Stop Cortaderia
- A través de las redes sociales de Stop Cortaderia
- En una formación de LIFE Stop Cortaderia
- A través de otras redes sociales
- A través de una actividad de voluntariado
- Durante una formación académica
- A través de familiares o amigos
- No me acuerdo
- El plumero no es una planta invasora
- Other: ____

11. Finalmente, y como desafío, ¿qué otra planta o plantas usarías para sustituir la Cortaderia como especie ornamental en los jardines? *

____

¡Gracias por su participación!

Para saber más sobre esta especie y su problemática

LIFE Stop Cortaderia:
stopcortaderia.org
facebook, instagram, twitter:
#stopcortaderia
stopcortaderiapt@gmail.com

This content is neither created nor endorsed by Google.

Google Forms
