# Supplementary material for: Public perceptions about the invasive pampas grass, Cortaderia selloana: a case study of environmentally conscious citizens in Southern Europe
Source: Biol Invasions. 2023 Mar 28;25(6):2043–56. doi: 10.1007/s10530-023-03025-3 (PMC10042667; doi:10.1007/s10530-023-03025-3)
Supplement: Supplementary file 3 — (PDF 129 KB) [file 10530_2023_3025_MOESM3_ESM.pdf]

Statistical results from the association between respondents´ profile data and their knowledge and perception about pampas grass

Table S1 Statistical results from the association between respondents' profile data and their basic knowledge of the plant: whether they recognize the plant (Q6) and if they know the plant´s name (Q6.1). *For these questions, Fisher´s Exact Test (FET) was used because assumptions of the Chi-Square test were not met. "-" means there´s no association between variables. For questions that showed a significant association, a graph of the responses is shown in Appendix 5.

Question	Respondents Profile	Portugal			Spain		
		Chi-square	Significance	Phi Cramer´s v	Chi-square	Significance	Phi Cramer´s v
Q6 Do you recognize the plant?	Q1 – Age	3.187*	0.140	-	9.230*	0.011	0,108 (small)
	Q2 – Gender	3.238	0.072	-	5.171	0.023	0,079 (small)
	Q3 – Education	2.505	0.286	-	5.263	0.072	-
	Q4 – Occupation	20.635*	0.003	0,214 (medium)	2.371*	0.938	-
	Q5 – Country	0.737*	0.682	-	2.506*	0.288	-
Q6.1 If yes, what´s the name of the plant?	Q1 – Age	11.310*	0.019	0.124 (small)	4.502*	0.469	-
	Q2 – Gender	3.157	0.206	-	13.885	0.003	0,135 (small)
	Q3 – Education	4.467*	0.287	-	0.856*	0.981	-
	Q4 – Occupation	31.369*	0.002	0,186 (small)	9.627*	0.745	-
	Q5 – Country	5.934*	0.152	-	13.843*	0.009	0.106 (small)

Table S2 Statistical results from the association between respondents´ profile data and their complex knowledge of the plant, related to the ecology and biology of the plant, through some statements (Q7). *For these questions, Fisher´s Exact Test (FET) was used because assumptions of the Chi-Square test were not met. "-" means there´s no association between variables. For questions that showed a significant association, a graph of the responses is shown in Appendix 5.

**Question**	**Respondents Profile**	**Portugal**			**Spain**		
		**Chi-square**	**Significance**	**Phi Cramer´s v**	**Chi-square**	**Significance**	**Phi Cramer´s v**
Q7 Select the statements that, in your opinion, are most appropriate for this plant	Q1 – Age	1.814*	0.757	-	18.545*	0.000	0.112 (small)
	Q2 – Gender	0.552	-0.759	-	12.824	0.002	0.130 (small)
	Q3 – Education	8.329*	0.059	-	2.196	0.700	-
	Q4 – Occupation	32.292*	0.002	0,197 (small)	19.224*	0.116	-
	Q5 – Country	0.937*	0.934	-	3.593*	0.605	-

Table S3 Statistical results from the association between respondents' profile data and their complex knowledge of the plant, related to the invasiveness of the plant and legislation questions (Q8, Q8.1, Q8.2). *For these questions, Fisher´s Exact Test was used because assumptions of the Chi-Square test were not met. "-" means there´s no association between variables. For questions that showed a significant association, a graph of the responses is shown in Appendix 5.

Question	Respondents Profile	Portugal			Spain		
		**Chi-square**	**Sig.**	**Cramer´s v**	**Chi-square**	**Sig.**	**Cramer´s v**
Q8 Is pampas grass an invasive plant in your country?	Q1 – Age	3.408*	0.186	-	26.351*	0.000	0.227 (medium)
	Q2 – Gender	2.768	0.096	-	1.800	0.180	-
	Q3 – Education	1.748	0.417	-	2.223	0.329	-
	Q4 – Occupation	24.221*	0.001	0,232 (medium)	16.431*	0.011	0,168 (small)
	Q5 – Country	1.331*	0.642	-	1.898*	0.408	-
Q8.1 If you answered yes to the previous question, do you know of any Decree-Law that limits its use?	Q1 – Age	1.409*	0.523	-	0.733	0.634	-
	Q2 – Gender	1.130	0.288	-	1.314	0.252	-
	Q3 – Education	12.513	0.002	0,179 (small)	12.124	0.002	0,131 (small)
	Q4 – Occupation	31.039*	<0.001	0,286 (medium)	5.721*	0.555	-
	Q5 – Country	2.211*	0.278	-	1.306*	0.573	-
Q8.2 If you answered yes to the previous question, which is this Decree-Law?	Q1 – Age	2.125*	0.382	-	0.792	0.684	-
	Q2 – Gender	0.378	0.828	-	0.443	0.801	-
	Q3 – Education	3.840*	0.511	-	10.247*	0.014	0,200 (medium)
	Q4 – Occupation	25.984*	0.008	0,328 (medium)	10.462*	0.732	-
	Q5 – Country	5.216*	0.242	-	1.616*	1.000	-

Table S4 Statistical results from the association between respondents' profile data and their complex knowledge of the pampas grass, related to the ecology and biology of the plant. *For these questions, Fisher´s Exact Test (FET) was used because assumptions of the Chi-Square test were not met. "-" means there´s no association between variables. For questions that showed a significant association, a graph of the responses is shown in Appendix 5.

**Question**	**Respondents Profile**	**Portugal**			**Spain**		
		**Chi-square**	**Significance**	**Cramer´s v**	**Chi-square**	**Significance**	**Cramer´s v**
Q9 In your opinion, what can be seen in the photos below?	Q1 – Age	2.154*	0.792	-	7.248*	0.087	-
	Q2 – Gender	0.163	0.922	-	4.768	0.092	-
	Q3 – Education	33.546*	0.000	0,199 (small)	4.461	0.347	-
	Q4 – Occupation	30.341*	0.003	0,188 (small)	16.198*	0.285	-
	Q5 – Country	10.219*	0.019	0.104 (small)	1.978*	0.817	-

Table S5 Statistical results from the association between respondents' profile data and their knowledge on Q10 – how did you know that this species is an invasive plant? The invasiveness of the plant was also analyzed. *For these questions, Fisher´s Exact Test (FET) was used because assumptions of the Chi-Square test were not met. "-" means there´s no association between variables. For questions that showed a significant association, a graph of the responses is shown in Appendix 5.

Question	Respondents Profile	Portugal			Spain		
		Chi-square	Significance	Cramer´s v	Chi-square	Significance	Cramer´s v
Q10 How did you know it is an invasive plant?	Q1 – Age	15.235*	0.826	-	26.086*	0.075	-
	Q2 – Gender	11.267*	0.149	-	13.659	0.135	-
	Q3 – Education	19.960*	0.193	-	25.661*	0.057	-
	Q4 – Occupation	75.027*	0.019	-	59.457*	0.381	-
	Q5 – Country	32.367*	0.008	0,278 (medium)	23.292*	0.643	-

Table S6 Statistical results from the association between respondents' profile data and their knowledge on Q11 – Finally, and as a challenge, instead of the pampas grass, what other plant(s) would you use as ornamentals in gardens? *For these questions, Fisher´s Exact Test (FET) was used because assumptions of the Chi-Square test were not met. "-" means there´s no association between variables. For questions that showed a significant association, a graph of the responses is shown in Appendix 5.

**Question**	**Respondents Profile**	**Portugal**			**Spain**		
		**Chi-square**	**Significance**	**Cramer´s v**	**Chi-square**	**Significance**	**Cramer´s v**
Q11 What other plant(s) would you use?	Q1 – Age	7.345*	0.477	-	9.247*	0.326	-
	Q2 – Gender	1.604*	0.820	-	3.237*	0.522	-
	Q3 – Education	6.749*	0.503	-	4.614*	0.749	-
	Q4 – Occupation	43.907*	0.009	0,152 (not sig)	15.039*	0.963	-
	Q5 – Country	25.022*	<0.001	0,178 (small)	9.414*	0.603	-
