# Supplementary material for: Public perceptions about the invasive pampas grass, Cortaderia selloana: a case study of environmentally conscious citizens in Southern Europe
Source: Biol Invasions. 2023 Mar 28;25(6):2043–56. doi: 10.1007/s10530-023-03025-3 (PMC10042667; doi:10.1007/s10530-023-03025-3)
Supplement: Supplementary file 4 — (PDF 684 KB) [file 10530_2023_3025_MOESM4_ESM.pdf]

Meaningful associations between respondents´ profile and their knowledge of the pampas grass

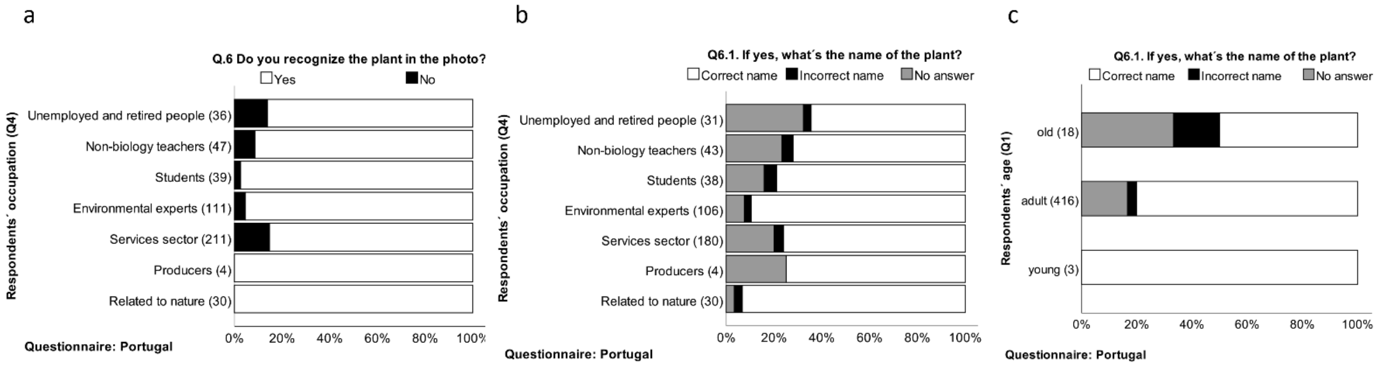

Fig. S1 Association between Portuguese respondent´s occupation (Q4) and whether they recognize the plant in the photo (Q6) (a) and know its name (Q6.1) (b), and between respondent´s age (Q1) and whether they know its name (Q6.1) (c). The values between parentheses represent the sample size (N).

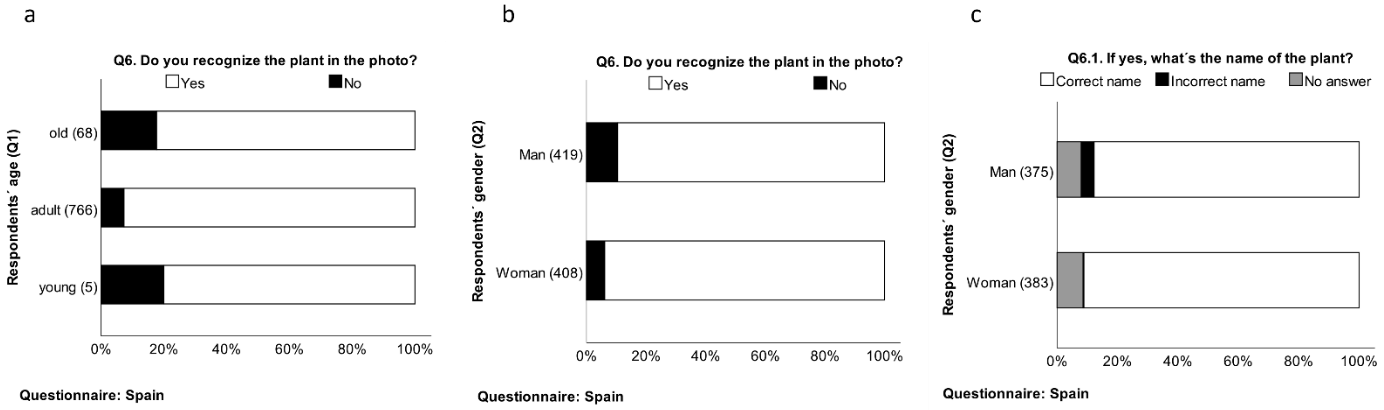

Fig. S2 Association between Spanish respondent´s age (Q1) and whether they recognize the plant in the photo (Q6) (a) and between respondent´s gender (Q2) and whether they recognize the plant in the photo (Q6) and know its name (Q6.1) (c). The values between parentheses represent the sample size (N).

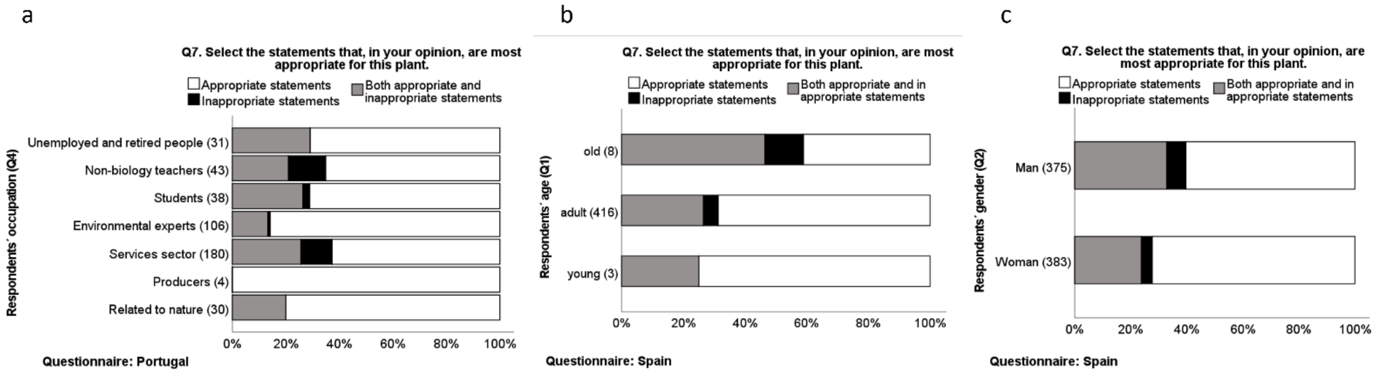

Fig. S3 Association between Portuguese respondent´s occupation (Q4) and their knowledge about the plant (Q7) (a) and between Spanish respondent´s age (Q1) (b) and gender (Q2) (c) and their knowledge about the plant (Q7). The original categories were rearranged in “more accurate”, “least accurate” and “both accurate and inaccurate” statements. The values between parentheses represent the sample size (N).

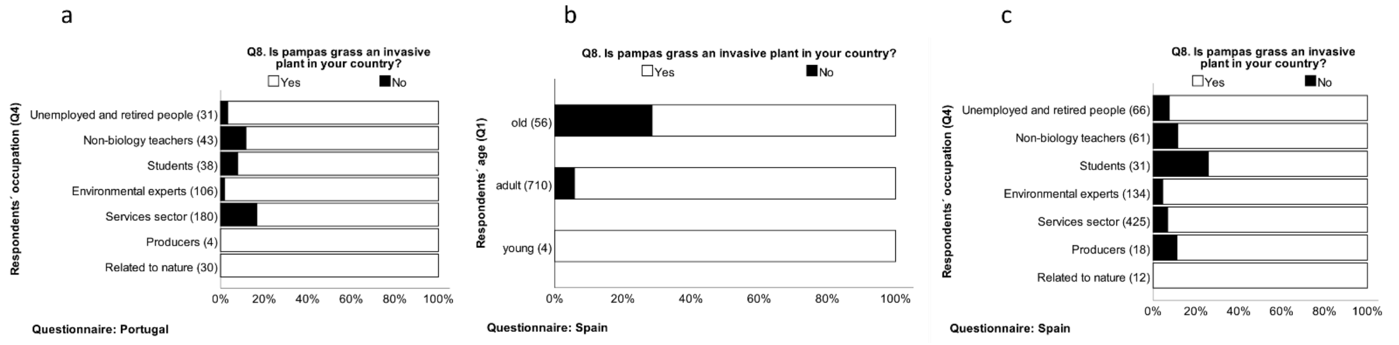

Fig. S4 Association between Portuguese respondent´s occupation (Q4) (a), and Spanish respondent´s age (Q1) (b) and occupation (Q4) (c) and knowing that pampas grass is invasive (Q8). The values between parentheses represent the sample size (N).

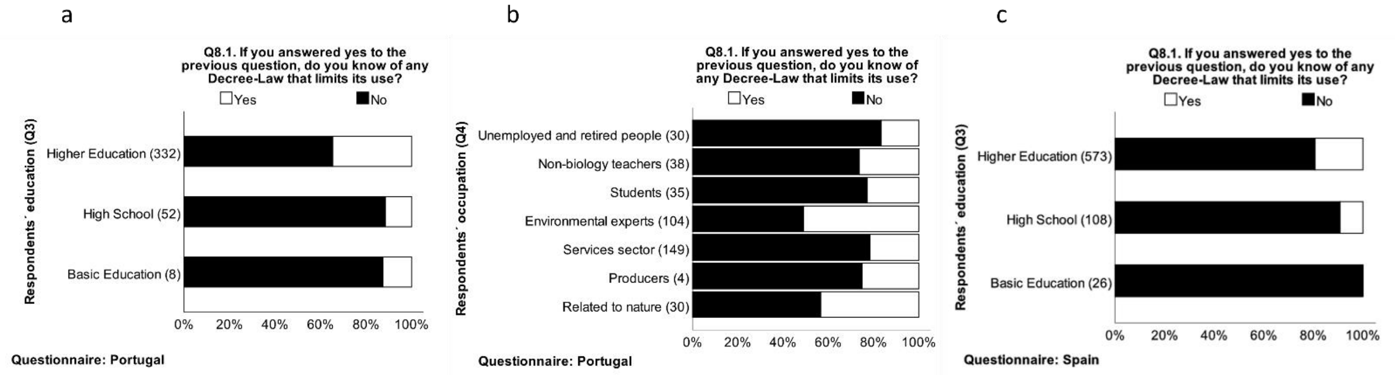

Fig. S5 Association between Portuguese respondent´s education (Q3) (a) and occupation (Q4) (b), and Spanish respondent´s education (Q3) (c) and whether they know of any Law that limits the use of pampas grass (Q8.1). The values between parentheses represent the sample size (N).

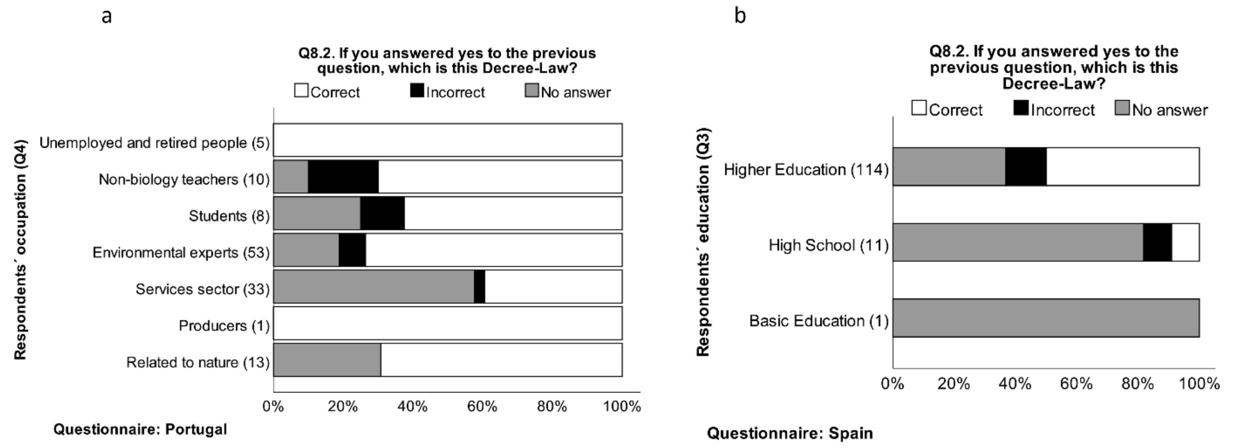

Fig. S6 Association between Portuguese respondent´s occupation (Q4) (a) and Spanish respondent´s education (Q3) (b) and whether they know the name of the Law that limits the use of Pampas grass (Q8.2). The values between parentheses represent the sample size (N).

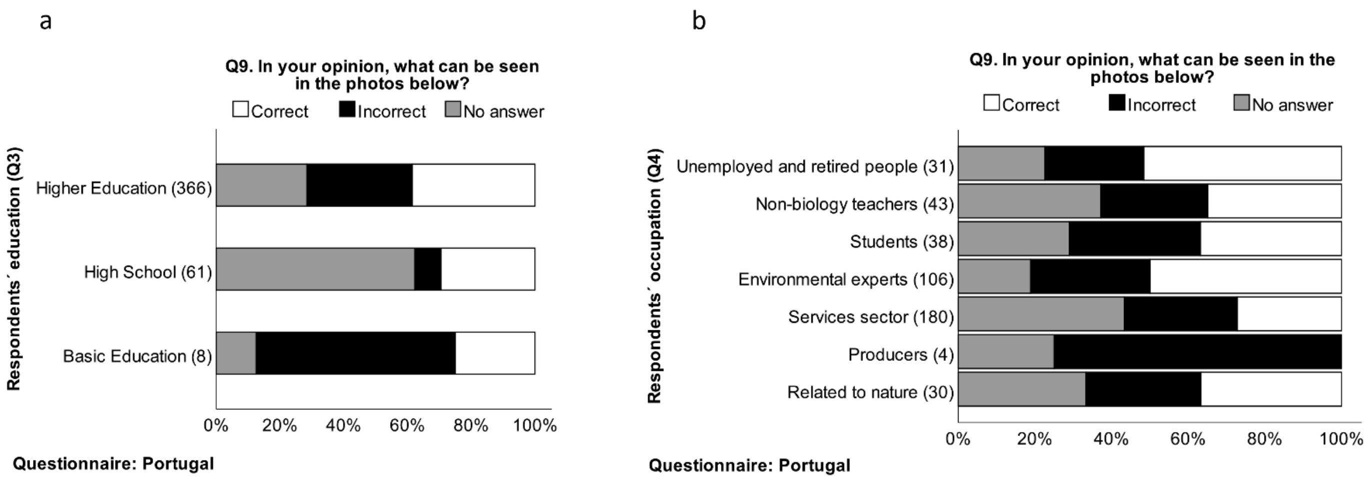

Fig. S7 Association between Portuguese respondent´s education (Q3) (a) and occupation (Q4) (b) with correctly identifying pampas grass photos (Q9). The values between parentheses represent the sample size (N).
